# Profiles and Networks: A Person‐Centered Analysis of Emotional Labor, Burnout, and Change Fatigue Among Chinese Oncology Nurses

**DOI:** 10.1155/jonm/4262055

**Published:** 2026-07-12

**Authors:** Hang Gao, Xiaoli Sun, Danwen Zheng, Hejia Chen, Xinyan Yu, Jianwen Hou

**Affiliations:** ^1^ Phase I Clinical Ward, Zhejiang Cancer Hospital, Hangzhou, China, zchospital.com; ^2^ Nursing Department, Zhejiang Cancer Hospital, Hangzhou, China, zchospital.com; ^3^ Head and Neck Surgery, Zhejiang Cancer Hospital, Hangzhou, China, zchospital.com

**Keywords:** burnout, change fatigue, computational simulated intervention, emotional labor, latent profile analysis, network analysis, nurse

## Abstract

**Aim:**

To identify distinct subtypes of emotional labor among Chinese oncology nurses; explore the heterogeneous network structures linking emotional labor, change fatigue, and burnout across subgroups; and explore potential intervention targets via computer simulation.

**Background:**

Oncology nurses face three key occupational challenges: inherent emotional labor demands, frequent organizational changes inducing change fatigue, and subsequent burnout risk. Although emotional labor is essential for quality care, its misregulation—especially the resource‐intensive deep acting—depletes psychological resources per conservation of resources theory. Yet, existing research often overlooks heterogeneity in nurses’ emotional labor strategy use.

**Methods:**

This study enrolled 924 nurses from a tertiary‐level specialized cancer hospital in Zhejiang Province, assessing them using scales for emotional labor, burnout, and change fatigue. First, latent profile analysis identified distinct subgroups based on emotional labor indicators. Subsequently, network analysis constructed symptom networks for the overall cohort and subgroups. Finally, computer simulation technology compared the effects of activating or inhibiting network nodes on overall symptom burden.

**Results:**

LPA revealed three profiles: “Efficient Type‐Proactive Regulation” (C1), “Stressed Type‐Natural Expression” (C2), and “Exhausted Type‐Dysregulated Regulation” (C3). In the total sample network, deep acting (E14) emerged as the most central and influential bridge symptom. Significant interprofile differences in network structure and global strength were observed, with unique core symptoms for each profile: professional efficacy (M10) for C1, overwhelming change (F1) for C2, and morning exhaustion (M3) for C3. Computer simulation identified potential core risk targets (M14, F1) and mitigation targets (E4, E8), with E8 showing cross‐profile robustness.

**Conclusion:**

Oncology nurses exhibit distinct emotional labor subtypes, and their burnout and change fatigue display significant, profile‐specific associative patterns. Simulation analysis highlights potential intervention targets: managing excessive work engagement and organizational evaluation pressure, while simultaneously promoting adaptive emotional regulation. This suggests that customized, systemic intervention strategies should be adopted, integrating individual skill development with organizational policy adjustments.

**Implications for Nursing Management:**

The findings of this study advocate for nursing managers to adopt evidence‐based, typology‐specific management strategies. For C1, professional recognition should be leveraged to enhance their sense of professional efficacy, while optimizing performance evaluations to reinforce adaptive regulation patterns. For C2, management should focus on buffering organizational change stress and leveraging the protective role of equitable evaluation through measures such as establishing flexible feedback mechanisms. For C3, immediate psychological support and short‐term leave resources are needed to alleviate physical and mental exhaustion and curb excessive work engagement. At the organizational level, systematically revising performance evaluation systems to reduce instrumental stress and embedding psychological resource support modules into change management processes is recommended. Concurrently, establishing a dynamic monitoring mechanism focused on core bridging symptom E14 facilitates early identification of high‐risk individuals.

## 1. Introduction

Emotion is a fundamental psychological process through which humans perceive the external world. The management of such emotions in occupational settings is termed emotional labor. This concept was first systematically proposed by sociologist Arlie Hochschild in her 1983 book The Managed Heart [1], referring to the process by which individuals regulate their internal and external emotional expressions to align with organizational norms, thereby achieving professional objectives. This phenomenon is particularly pronounced in nursing. Chinese nurses commonly experience moderate to high levels of emotional labor [2], with oncology nurses facing significantly greater emotional labor intensity than general wards due to frequent encounters with patients’ death anxiety, family emotional outbursts, and communication about treatment uncertainties. Moderate emotional labor investment can enhance work well‐being by boosting positive affect and optimizing nurse–patient relationships [3]. However, inappropriate expression strategies may lead to excessive resource depletion among nursing staff, exacerbating negative affect accumulation and triggering chain reactions such as physical and mental exhaustion, decreased job satisfaction, and diminished care quality [4–6]. The academic community widely employs Ashforth et al.’s three‐dimensional model to analyze emotional labor strategies [7]: surface acting (masking genuine emotions through feigned expressions and body language), deep acting (internalizing organizational emotional norms to assimilate feelings), and the expression of naturally felt emotions (also referred to as genuine affect or natural emotional expression, which involves unadjusted, authentic emotional displays when internal feelings naturally align with organizational expectations). In clinical practice, when nurses’ natural emotions conflict with professional demands, surface acting may temporarily maintain professional image but risks psychological exhaustion [4]. In contrast, deep acting facilitates emotional adaptation through cognitive restructuring, better sustaining positive work states [3]. The conservation of resources (COR) theory provides a critical explanatory framework. This theory defines “resources” as the various elements individuals require to achieve goals, emphasizing that initial resource levels directly influence subsequent resource investment capacity [8]. Without sufficient resource support, prolonged emotional labor among nurses leads to declining well‐being and work performance, creating a vicious cycle of stress and burnout [9]. Therefore, establishing an emotion management mechanism based on resource replenishment emerges as a vital pathway to enhance nursing professionals’ well‐being.

Meanwhile, global healthcare systems are undergoing rapid and continuous transformation [10]. In China, this transformation manifests as multidimensional hospital reforms encompassing technological upgrades, payment system reforms, departmental restructuring, and scale expansion [11, 12]. Although these changes aim to enhance medical efficiency and service quality, their high frequency and persistent nature subject nurses to immense adaptive pressure, leading to a state termed change fatigue. From an organizational behavior perspective, change fatigue refers to persistent exhaustion resulting from the continuous depletion of psychological resources under high‐intensity, high‐frequency organizational change pressures. Its core manifestations include emotional detachment, reduced willingness to participate in change, diminished professional identity, and impaired self‐efficacy perceptions [13, 14]. Research indicates that the incidence of change fatigue among nursing staff is approximately three times higher than among other healthcare professionals [15, 16]. This phenomenon not only directly exacerbates professional burnout, reduces job satisfaction, and increases talent attrition but also hinders the coordinated advancement of reforms, ultimately threatening the achievement of healthcare institutions’ overall strategic objectives. Currently, the cycle of “demand‐driven change⟶change‐induced stress⟶accumulated stress leading to fatigue” has become a critical bottleneck constraining the sustainable development of nursing teams.

Occupational burnout refers to a multidimensional psychological exhaustion state triggered by prolonged work pressure and interpersonal strain, primarily characterized by emotional exhaustion, depersonalization, and reduced personal accomplishment [17]. Recognized as an occupational disorder, this syndrome can induce mental health issues such as anxiety and depression. Among healthcare professionals, nurses are particularly vulnerable, with their high‐risk status stemming from the convergence of sociodemographic variables (gender, age, marital status, number of children), psychological variables (personality trait differences), and work‐related variables (work environment, compensation, colleague relationships, job satisfaction) [18–21]. Oncology nurses face heightened susceptibility due to their unique work characteristics: frequent exposure to death‐related contexts (regularly managing patient suffering/death and family grief), complex conflict environments (navigating multilevel conflicts—both organizational [colleagues/managers] and clinical [patients/families]), and emotional overload (sustained emotional investment in intricate interpersonal interactions). These factors further increase oncology nurses’ vulnerability to burnout, ultimately compromising care quality, reducing work productivity, and impairing clinical focus [22]. Empirical evidence further underscores burnout’s detrimental effect on end‐of‐life care, emphasizing the urgent need for targeted intervention [23].

In summary, oncology nurses commonly face high incidence, high intensity, and multifactorial occupational burnout. This phenomenon is closely linked to persistent resource depletion caused by high‐intensity emotional labor and change fatigue triggered by frequent organizational transformations. These factors intertwine and reinforce each other, forming a complex network system that demands in‐depth analysis. Existing studies predominantly rely on total scale scores to assess emotional labor levels, failing to adequately reflect heterogeneity within the population. To address this limitation, this study introduces latent profile analysis (LPA)—an individual‐centered approach [24], to identify latent subgroups through response patterns of manifest variables, thereby revealing differentiated combinations of emotional labor strategies among oncology nurses. Building upon this, network analysis is further employed by abstracting variables as nodes and relationships as edges, combined with centrality metrics to identify key nodes [25, 26] to dissect the interactive mechanisms among key variables within each subgroup. This study integrates the research pathway of “LPA and network analysis” to systematically compare the network characteristics of change fatigue and occupational burnout among Chinese oncology nurses under varying emotional labor intensities. It aims to provide evidence for developing targeted, evidence‐based interventions that support nurses’ psychological resilience, alleviate occupational burnout, and ultimately contribute to building a sustainable, high‐quality oncology nursing workforce.

## 2. Materials and Methods

### 2.1. Study Design and Participants

This cross‐sectional study was reported in accordance with the Strengthening the Reporting of Observational Studies in Epidemiology (STROBE) guidelines for cross‐sectional studies. This cross‐sectional study was conducted at a specialized cancer hospital in Hangzhou, China, from January 2024 to April 2025. Inclusion criteria were registered nurses with over one year of work experience who were currently employed and voluntarily signed informed consent forms. Exclusion criteria included history of mental illness or cognitive impairment or experiencing major psychologically traumatic events within the past 6 months (e.g., bereavement of spouse or child, diagnosis of malignant tumors or other severe illnesses). Sample size was estimated using Kline’s recommended method, which suggests a sample size of 10–20 times the number of variables, plus an additional 20% allowance for invalid questionnaires. Based on this, the study required a sample size of approximately 840 participants.

Data were collected via electronic questionnaires, with the introductory section detailing the study’s objectives, significance, and completion guidelines. All questions were configured as mandatory after logical branching, and each IP address was restricted to a single submission. Consequently, there were no item‐level missing data, precluding the need for missing data imputation in the subsequent LPA and network analyses. Research team members distributed the questionnaire QR code, and participating nurses completed and submitted it anonymously after providing informed consent. After collection, two reviewers independently verified the questionnaires, excluding invalid responses based on the following criteria: completion time under 5 min (the shortest estimated completion time from a presurvey), answers exhibiting obvious patterns, or logical inconsistencies. This study adheres to the principles outlined in the Declaration of Helsinki and received approval from the Medical Ethics Committee of Zhejiang Cancer Hospital, with Ethics Approval Number IRB‐2023‐973.

### 2.2. Demographic Information

Designed by the research team, the questionnaire includes information on gender, age, marital status, educational background, years of work experience, number of night shifts per month, employment status, professional title, job position, department, and personality traits.

### 2.3. Emotional Labor

The Chinese version of the Emotional Labor Scale for Nurses was revised from the original scale developed by Luo Hong et al. [27]. This version incorporates the clinical nursing practice context in China and comprises three dimensions: surface acting, deep acting, and expression of naturally felt emotions, totaling 14 items. The scale employs a 6‐point scoring method (1–6 points), with total scores ranging from 14 to 84 points. Higher scores indicate greater emotional labor burden. Its clear and easily understandable items demonstrate good applicability and widespread use among Chinese nursing populations [28]. In this study, the scale achieved Cronbach’s α coefficient of 0.850.

### 2.4. Burnout

In this study, nurses’ levels of occupational burnout were measured using the Chinese version of the Maslach Burnout Inventory—General Survey (MBI‐GS). Developed by Maslach [29] and adapted and revised for Chinese contexts by scholars Li and Shi [30], this scale demonstrates strong applicability and a broad foundation of use within Chinese settings. The scale comprises 15 items covering three dimensions: emotional exhaustion, depersonalization, and reduced personal accomplishment. It employs a 7‐point scoring scale ranging from 0 (never) to 6 (daily), with items in the personal accomplishment dimension reverse‐scored and the remaining dimensions scored normally. Total scores range from 0 to 90, with higher scores indicating more severe burnout. In this study, Cronbach’s α coefficient for this scale was 0.877.

### 2.5. Change Fatigue

The Change Fatigue Scale (CFS) was developed by Bernerth et al. [13] in 2011 to assess employees’ fatigue resulting from organizational change. This scale was adapted and revised for the Chinese context by Zhang et al. [31], making it suitable for measuring change fatigue among Chinese nursing staff. The Chinese version comprises six items consistent with the original scale structure, employing a 7‐point Likert scale ranging from “*Strongly Disagree*” (1 point) to “*Strongly Agree*” (7 points). Higher total scores indicate greater fatigue experienced by employees during organizational change processes. In this study, the scale demonstrated Cronbach’s α coefficient of 0.852.

### 2.6. Statistical Analyses

**FIGURE 1:**
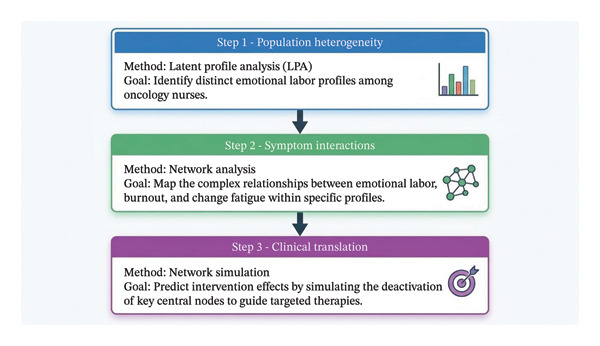
The sequential three‐step analytical framework of the current study.

To manage analytical complexity and provide a clear, logical structure, our study employed a sequential, three‐step analytical framework (illustrated in Figure 1). In Step 1, we utilized LPA to identify distinct subgroups of nurses based on their emotional labor characteristics, thereby capturing population heterogeneity. In Step 2, we applied network analysis to these specific subgroups to map the complex, interacting relationships among emotional labor, change fatigue, and burnout symptoms. Finally, in Step 3, we conducted network‐based simulations on these estimated network structures to predict the potential outcomes of targeting specific core symptoms. This step‐by‐step approach allows us to translate population classification into interaction mechanisms and ultimately into practical clinical intervention strategies.

Statistical analysis was performed using SPSS 25.0 and R software. Normally distributed quantitative data are presented as mean ± standard deviation ($\overset{¯}{x}$ ± s), whereas categorical data are described as frequency and percentage (%). Intergroup comparisons employed analysis of variance (ANOVA), *X* ^2^ test, or Fisher’s exact test based on data type and conditions. Multivariate logistic regression analyzed factors influencing different dimensions of nurses’ emotional labor, with a significance level of α = 0.05.

First, LPA was conducted on nurses’ emotional labor using the poLCA package in R 4.3.2. Starting with one latent category, the number of categories was incrementally increased. The optimal model was determined by comparing the fit indices of different category models. Model fit evaluation metrics included Akaike information criterion (AIC), Bayesian information criterion (BIC), and adjusted BIC (aBIC), where lower values indicate better model fit; entropy is used to assess classification accuracy, with values ranging from 0 to 1, where values closer to 1 indicate greater classification precision. The likelihood ratio test (LRT) and bootstrap LRT (BLRT) are employed to compare the fitting differences among models with varying numbers of categories. If *p* < 0.05, it indicates that the *k*‐category model is significantly superior to the *k* − 1‐category model [32, 33].

Second, a heatmap was generated using the corrplot package in R 4.3.2 software to analyze the correlations among emotional labor, occupational burnout, and change fatigue. The intensity of colors in the heatmap reflects the similarity or distance between clusters: The gradient scale on the right indicates the magnitude of correlation coefficients, where white represents 0, darker blue indicates values closer to 1, and darker red indicates values closer to −1. The size and color intensity of each circle collectively represent both the magnitude and direction of the correlation coefficient, with larger circles indicating stronger correlations (closer to 1 or ‐1) [34].

Finally, a visual network was constructed using the “qgraph” package in R 4.3.2 software, with symptom networks mapped based on the EBICglasso algorithm and Spearman’s correlation analysis. Based on items from the Emotional Labor Scale, Burnout Scale, and CFS, a synchronous network comprising 36 symptoms was constructed. The network underwent sparsification using the least absolute and selective optimization (LASSO) algorithm to yield a streamlined structure. Nodes in the network represent symptoms corresponding to each item, whereas edges denote conditional independence relationships between symptoms, with edge thickness reflecting relationship strength. The centrality plot function in the qgraph package calculates and visualizes node centrality metrics, including strength centrality, closeness centrality, and betweenness centrality [35]. Strength centrality (the sum of absolute edge weights connected to a node) was selected as the primary centrality metric because it is the most stable and reliable index in cross‐sectional network analysis [36, 37]. Betweenness and closeness centrality rely on assumptions (e.g., presence of a flow and shortest paths) that may not correspond to how psychological variables relate to one another [36] and were therefore considered secondary. Higher strength centrality values indicate greater importance within the network. Bridge centrality metrics assess a node’s role as a bridge connecting different symptom clusters, encompassing bridging strength, bridging proximity, and bridging intermediary. For bridge symptoms, bridge strength was used as the primary metric for analogous reasons. The bootnet package was employed to test the robustness of the network model [26], calculating the correlation stability (CS) coefficient via case‐dropping bootstrap. CS > 0.25 indicates good stability of the centrality metrics. The Network Comparison Test package was used to perform hypothesis tests for structural invariance, invariance of global strength, and invariance of centrality across different gender subgroups [38], with a significance level set at *α* = 0.05.

In this study, we employed the R packages “bootnet,” “qgraph,” and “nodeIdentifyR” to perform model estimation and data simulation. Based on the estimated parameters extracted from the model, we generated 15,000 simulated “participants.” We then applied two types of interventions to the network: alleviating interventions and aggravating interventions. Following the NodeIdentifyR algorithm [39], the intervention magnitude was set to two standard deviations of the estimated threshold parameters. In particular, after estimating the network model, we extracted the thresholds for all symptoms, computed their standard deviation, and used this value to adjust the threshold parameters of each symptom individually. This standardized perturbation (rather than a fixed absolute value) ensures comparability of intervention effects across symptoms with different baseline thresholds, given the nonlinear nature of the model. The algorithm simulates network behavior under adjusted parameters, calculates changes in overall symptom activation levels relative to the baseline model, and identifies nodes that are estimated to exert the most significant predicted impact on network behavior within the model’s structure [40].These findings are model‐derived projections (in silico trials) and should be interpreted as exploratory, and empirical validation through longitudinal or experimental research is required.

## 3. Results

### 3.1. Latent Profiles of Emotional Labor

**FIGURE 2:**
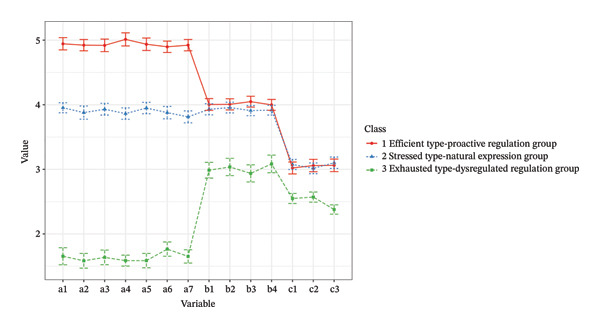
Potential profile distribution characteristics of nurses’ emotional labor (a1–a7 represents seven entries in the surface acting dimension of emotional labor, b1–b4 represents four entries in the natural expression dimension, and c1–c3 represents three entries in the deep acting dimension).

In this study, we used the 14 items of the “Emotional Labor Scale” as manifest variables and employed LPA to cluster nurses’ emotional labor patterns. We estimated models containing 1 to 5 latent profiles. Among these, models with 1 to 3 classes successfully converged, with their fit indices shown in Table 1. As the number of classes increased from 1 to 3, the AIC, BIC, and SABIC values decreased continuously, and the entropy values all exceeded 0.80. We also systematically compared four common covariance structures (VVV, VVI, VEI, and EII) using BIC; the VVI model produced the lowest BIC (33,040.62) for the 3‐class solution and was therefore selected as the optimal covariance structure (see Supporting Table S1). We further attempted to estimate 4‐ and 5‐class models; however, due to a sharp increase in the number of parameters, the EM algorithm failed to converge (manifested as class membership probabilities tending toward extreme values or a singular covariance matrix), and thus, no valid solutions were obtained. The BLRT indicated that both the 2‐class and 3‐class models were significantly superior to their preceding models (*p* < 0.01). Although the 2‐class solution also exhibited good statistical fit, its population distribution was highly uneven; in contrast, the 3‐class solution divided nurses into three subgroups with more balanced proportions, better reflecting the heterogeneity of emotional labor. For the final 3‐class solution, the average posterior probability ranged from 93.5% to 99.9%, indicating high classification reliability. We further assessed the local independence assumption by inspecting the standardized residual correlation matrix within the latent profiles. The median absolute residual correlation was 0.034, and all values were below 0.20 (maximum absolute *r* = 0.125), indicating that the majority of variable associations were adequately explained by the latent model. Only 1.1% of the pairwise correlations exceeded 0.10, and none exceeded 0.20, fully supporting the local independence assumption. The complete posterior probability matrix for the three‐class solution has been uploaded as Supporting Information (see Supporting Table S1). Figure 2 illustrates the feature distributions of the three emotional labor profiles. Based on statistical results and clinical interpretability, we named these three profiles as follows: C1: “Efficient Type‐Proactive Regulation Group” (*n* = 369, 39.9%); C2: “Stressed Type‐Natural Expression Group” (*n* = 415, 44.9%); and C3: “Exhausted Type‐Dysregulated Regulation Group” (*n* = 140, 15.2%).

**TABLE 1:** Indicators for fitting a potential profile model of nurses’ emotional labor.

Classes	LogLike	AIC	BIC	SABIC	Entropy	BLR_p	Proportion	AvgPosterior
1	−19346.8	38,749.6	38,884.8	38,795.8	1.000	—	—	—
2	−16715.3	33,544.5	33,819.8	33,638.7	0.996	< 0.01	0.844/0.156	0.999/0.995
**3**	**−16226.7**	**32,625.4**	**33,040.6**	**32,767.5**	**0.889**	**<** **0.01**	**0.399/0.449/0.152**	**0.935/0.944/0.999**

*Note:* 4 and 5 class solutions were attempted but failed to converge. Entropy is not applicable to the 1‐class solution. Average posterior probabilities are based on the posterior classification matrix diagonal elements. For the 2‐class solution, these values are 0.999 and 0.995, indicating very high classification certainty; for the 3‐class solution, the values range from 0.935 to 0.999, still indicating good separation. The bold values in indicate the key statistical indices corresponding to the three selected latent profiles in this study.

### 3.2. Participant Characteristics and Profile Differences

**FIGURE 3:**
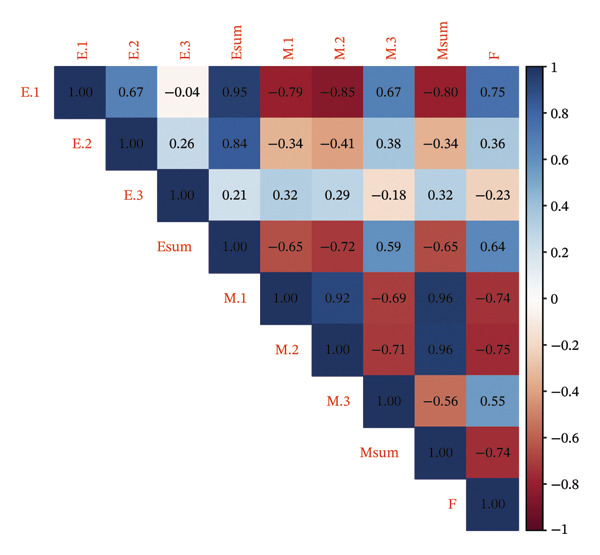
Heatmap of correlations between emotional labor, burnout, and change fatigue (E1: surface acting; E2: expression of naturally felt emotions; E3: deep acting; Esum: emotional labor total score; M1: emotional exhaustion dimension; M2: dehumanization dimension; M3: reduced personal accomplishment dimension; Msum: burnout total score; F: change fatigue total score).

A total of 985 questionnaires were distributed, and 61 invalid responses were removed (36 due to completion time under 5 min, 17 due to obvious response patterns, and 8 due to logical inconsistencies), yielding a final sample of 924 valid responses (a valid response rate of 93.8%), with 83.1% female. Detailed demographic characteristics and univariate comparisons across the three latent profiles are presented in Table 2. Significant differences across profiles were observed in gender, age, marital status, education, work experience, night shifts, employment type, job title, department, and personality traits (exact *p*‐values in Table 2). The variables found to be statistically significant in these univariate analyses were then included in the logistic regression model as covariates to adjust for potential confounding effects. Multivariate logistic regression showed that age, work experience, night shifts, job title, work unit, neuroticism, and conscientiousness independently predicted profile membership (Table 3). Moreover, the three profiles differed significantly in total burnout and change fatigue scores, as well as in all subdimensions (emotional exhaustion, depersonalization, reduced personal accomplishment; all *p* < 0.001; Table 4). Partial correlation analysis, adjusting for the significant demographic variables, revealed the relationships among burnout, emotional labor, and change fatigue (Figure 3). Specifically, surface acting was positively associated with change fatigue but negatively associated with emotional exhaustion and depersonalization, whereas deep acting showed the opposite pattern.

**TABLE 2:** General profiles of nurses and their differences in potential profiles of emotional labor (*n* = 924).

Items		*N* (%)	C1: Efficient Type‐Proactive Regulation Group (*n* = 369, 39.9%)	C2: Stressed Type‐Natural Expression Group (*n* = 415, 44.9%)	C3: Exhausted Type‐Dysregulated Regulation Group (*n* = 140, 15.2%)	*X* ^2^	*p*
Gender	Female	788 (85.3)	326	331	131	20.510^a^	< 0.001
	Male	136 (14.7)	43	84	9		

Age (years)	≤ 30	211 (22.8)	56	58	91	217.705^a^	< 0.001
	31–40	314 (34)	145	135	34		
	≥ 41	399 (43.2)	168	222	15		

Marital status	Unmarried	372 (40.3)	107	194	71	53.916^a^	< 0.001
	Married	456 (49.4)	231	181	44		
	Others	96 (10.4)	31	40	25		

Education level	High school and below	349 (37.8)	103	127	119	189.066^a^	< 0.001
	Undergraduate	523 (56.6)	226	281	16		
	Masters and above	52 (5.6)	40	7	5		

Years of experience	≤ 5	225 (24.4)	75	78	72	94.347^a^	< 0.001
	6–10	281 (30.4)	126	107	48		
	≥ 11	418 (45.2)	168	230	20		

Night shifts per month	≤ 4	317 (34.3)	89	189	39	118.430^a^	< 0.001
	5–10	417 (45.1)	232	145	40		
	≥ 11	190 (20.6)	48	81	61		

Employment type	Officially on board	762 (82.5)	330	360	72	155.391^a^	< 0.001
	Personnel agent	125 (13.5)	21	39	65		
	Contract system	37 (4.0)	18	16	3		

Job title	Junior	371 (40.2)	153	148	70	28.287^a^	< 0.001
	Middle	446 (48.3)	180	224	42		
	Senior	107 (11.6)	36	43	28		

Workplace	Clinical nursing post	785 (85)	307	351	127	4.571^a^	0.102
	Nursing management post	139 (15)	62	64	13		

Department	General medicine	294 (31.8)	99	175	20	111.639^a^	0.049
	Neurosurgery	245 (26.5)	82	116	47		
	Emergency/operating room/ICU	206 (22.3)	90	52	64		
	Others	179 (19.4)	98	72	9		

Personality traits	Neuroticism	107 (11.6)	14	21	72	308.421^a^	< 0.001
	Extraversion	301 (32.6)	162	117	22		
	Attractiveness	404 (43.7)	157	228	19		
	Dutifulness	47 (5.1)	13	19	15		
	Openness	65 (7.0)	23	30	12		

*Note:* The superscript “a” indicates that 0 cells have an expected count of less than 5, which means the Pearson chi‐square test assumption was fully satisfied for all variables marked with “a.”

**TABLE 3:** Multifactorial analysis of potential profiles of emotional labor in nurses.

Items		C1: Efficient Type‐Proactive Regulation Group					C2: Stressed Type‐Natural Expression Group				
		β	SE	*p*	OR	95% CI	β	SE	*p*	OR	95% CI
Age (years)	< 30	9.681	2.022	< 0.001	—	—	7.809	2.063	< 0.001	—	—
	31–40	−6.780	0.949	< 0.001	0.001	0.000–0.007	−6.419	0.937	< 0.001	0.002	0.000–0.010
	≥ 41^∗^	−2.985	0.745	< 0.001	0.051	0.012–0.218	−2.517	0.745	< 0.001	0.081	0.019–0.348

Gender	Female	−1.124	0.838	0.180	0.325	0.063–1.678	−0.685	0.827	0.408	0.504	0.100–2.551
	Male^∗^										

Marital status	Unmarried	−4.108	0.937	< 0.001	0.016	0.003–0.103	−4.047	0.916	< 0.001	0.017	0.003–0.105
	Married	−0.413	0.871	0.636	0.662	0.120–3.650	−0.296	0.850	0.727	0.743	0.141–3.934
	Others^∗^										

Education level	High school and below	−2.848	1.067	0.008	0.058	0.007–0.470	0.332	1.119	0.766	1.394	0.156–12.494
	Undergraduate	−1.472	1.104	0.183	0.230	0.026–1.999	3.584	1.159	0.002	36.015	3.715–349.163
	Masters and above^∗^										

Years of experience	≤ 5	−2.750	0.802	0.001	0.064	0.013–0.308	−4.086	0.787	< 0.001	0.017	0.004–0.079
	6–10	1.465	0.826	0.076	4.326	0.857–21.851	−1.755	0.788	0.026	0.173	0.037–0.810
	≥ 11^∗^										

Night shifts per month	≤ 4	5.717	0.901	< 0.001	303.864	51.969–1776.690	5.399	0.890	< 0.001	221.219	38.648–1266.265
	5–10	7.325	0.914	< 0.001	1517.102	252.733–9106.822	2.439	0.846	0.004	11.462	2.185–60.115
	≥ 11^∗^										

Employment type	Officially on board	−0.956	1.248	0.444	0.385	0.033–4.435	−0.361	1.238	0.771	0.697	0.062–7.896
	Personnel agent^∗^	−4.266	1.212	< 0.001	0.014	0.001–0.151	−2.353	1.158	0.042	0.095	0.010–0.920

Job title	Junior	0.947	0.836	0.258	2.577	0.500–13.273	1.726	0.846	0.041	5.620	1.071–29.500
	Middle	1.401	0.715	0.050	4.058	0.999–16.477	2.052	0.735	0.005	7.782	1.844–32.845
	Senior^∗^										

Department	General medicine	2.499	1.119	0.025	12.171	1.359–109.032	3.283	1.102	0.003	26.647	3.074–230.960
	Neurosurgery	−3.234	0.722	< 0.001	0.039	0.010–0.162	−2.343	0.710	0.001	0.096	0.024–0.387
	Emergency/operating room/ICU	−2.831	0.819	0.001	0.059	0.012–0.294	−4.571	0.828	< 0.001	0.010	0.002–0.052
	Others^∗^										

Personality traits	Neuroticism	−5.677	1.164	< 0.001	0.003	0.000–0.034	−4.589	1.076	< 0.001	0.010	0.001–0.084
	Extraversion	−1.462	1.002	0.145	0.232	0.033–1.653	−2.011	1.001	0.044	0.134	0.019–0.951
	Attractiveness	−0.605	0.949	0.524	0.546	0.085–3.508	−1.606	0.932	0.085	0.201	0.032–1.248
	Dutifulness	−6.008	1.208	< 0.001	0.002	0.000–0.026	−4.434	1.186	< 0.001	0.012	0.001–0.121
	Openness^∗^										

*Note:* C3: Exhausted Type‐Dysregulated Regulation Group is the reference group.

^∗^is the reference group.

**TABLE 4:** Differences in change fatigue and burnout among nurses with different potential profiles of emotional labor (*n* = 924).

Group	*N* (%)	Change fatigue	Emotional exhaustion	Depersonalization	Reduced personal accomplishment	Burnout
C1	369 (39.9%)	22.39 ± 1.855	18.38 ± 4.493	14.8 ± 4.624	17.02 ± 6.663	50.2 ± 5.074
C2	415 (44.9%)	23.38 ± 4.585	15.04 ± 1.853	9.7 ± 3.348	26.31 ± 6.057	51.05 ± 4.472
C3	140 (15.2%)	30.59 ± 5.347	15.24 ± 1.935	8.7 ± 2.006	29.99 ± 2.757	53.93 ± 3.304
F		5743.599	2605.390	4659.658	7020.406	901.780
P		< 0.001	< 0.001	< 0.001	< 0.001	< 0.001

*Note:* C1: Efficient Type‐Proactive Regulation Group; C2: Stressed Type‐Natural Expression Group; C3: Exhausted Type‐Dysregulated Regulation Group.

### 3.3. The Network Structure of Emotional Labor, Change Fatigue, and Burnout

**FIGURE 4:**
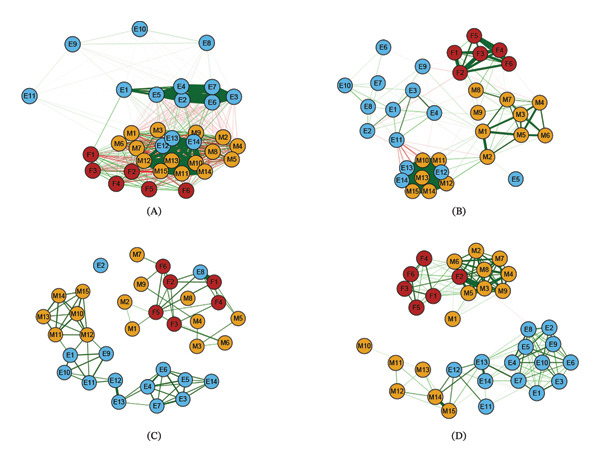
Symptom network in the total sample and three subgroups (the green line represents positive correlation, and the red line represents negative correlation). (A) Network analysis of the total sample. (B) Network analysis of the C1 sample. (C) Network analysis of the C2 sample. (D) Network analysis of the C3 sample.

Figure 4 displays the symptom networks for the total sample (A) and three subgroup profiles (B/C/D), presenting network analyses of emotional labor, occupational burnout, and change fatigue across the entire sample and three subgroups. Blue represents emotional labor, yellow represents occupational burnout, and red represents change fatigue. Networks were estimated using the EBICglasso method with a tuning parameter *γ* = 0.5. The symptom network for the total sample comprises 35 nodes and 279 edges (sparsity = 0.52, i.e., proportion of zero edges). Green lines indicate positive correlations, whereas red lines denote negative correlations. Based on edge thickness in the symptom network, E14 (“I make an effort to feel and embody the emotions I should express to patients”; rs = 15.928) emerges as the most central symptom within the overall network linking emotional labor, change fatigue, and occupational burnout. It is followed by E13 (When feeling down, I temporarily set aside my unpleasant mood for work purposes to ensure I can face patients with a positive attitude; rs = 15.625) and E12 (During work, I strive to overcome my own negative emotions and genuinely serve patients with warmth and kindness; rs = 15.223). Notably, all three belong to deep role‐playing strategies. The symptom pair with the strongest correlation in the total sample network is E4 (When facing patients, I project the required professional demeanor without altering my actual inner feelings) and E6 (I merely feign the emotions required for my role) (*r* = 0.792). For the subgroup networks, sparsity values were as follows: Profile C1 = 0.63, Profile C2 = 0.74, and Profile C3 = 0.68. The most central symptom in the C1 profile is M10 (I can effectively resolve issues that arise at work; rs = 7.450). The most central symptom for Profile C2 is F1 (The organization has introduced too many changes in certain areas; rs = 3.393), whereas Profile C3’s core symptom is M3 (Waking up in the morning and facing another day of work makes me feel incredibly tired; rs = 5.654). Entries for emotional labor, burnout, and change fatigue, along with their corresponding serial numbers, are listed in Table 5. For detailed information on the weights of each connection in the network, please refer to Supporting Information: Supporting Table S2, Supporting Table S3, Supporting Table S4, and Supporting Table S6.

**TABLE 5:** Entries and numbers for emotional labor, burnout, and change fatigue.

Scale	Entries	
Emotional labor	E1: Displaying appropriate expressions and attitudes at work feels like acting to me	E2: The emotions I show to patients differ from what I truly feel inside
	E3: To serve patients appropriately, I’ve learned to fake emotions	E4: When facing patients, I project the required professional demeanor without altering my actual inner feelings
	E5: To project specific expressions and attitudes toward patients, I conceal my genuine emotions	E6: I merely feign the emotions required for my role
	E7: My job demands displaying emotions that diverge from my authentic self	E8: The hospital evaluates my work attitude as part of performance reviews
	E9: The hospital mandates specific behaviors or facial expressions to project its desired image	E10: The hospital requires me to adopt different emotional responses for different patients
	E11: When patients make unreasonable demands, the hospital requires me to maintain a warm and friendly demeanor	E12: During work, I strive to overcome my own negative emotions and genuinely serve patients with warmth and kindness
	E13: When feeling down, I temporarily set aside my unpleasant mood for work purposes to ensure I can face patients with a positive attitude	E14: I make an effort to feel and embody the emotions I should express to patients

Maslach burnout	M1: My job leaves me physically and mentally drained	M2: By the time I clock out, I feel utterly exhausted
	M3: Waking up in the morning and facing another day of work makes me feel incredibly tired	M4: Working all day is genuinely stressful for me
	M5: Work makes me feel like I’m about to break down	M6: Since starting this job, I’ve become increasingly disinterested in my work
	M7: I’m not as enthusiastic about my job as I used to be	M8: I question the meaning of the work I do
	M9: I care less and less about whether my work makes a difference	M10: I can effectively resolve issues that arise at work
	M11: I feel I’m making a useful contribution to the company	M12: In my view, I excel at my job
	M13: I feel very pleased when I accomplish tasks at work	M14: I complete a lot of valuable work
	M15: I’m confident I can effectively handle all my responsibilities	

Change fatigue	F1: The organization has introduced too many changes in certain areas	F2: I’m tired of all the changes within the organization
	F3: The changes occurring in certain areas are substantial	F4: We’re being asked to alter too many things in certain aspects
	F5: It feels like we’re constantly being asked to change something	F6: When the organization implements changes, I wish for a period of stability to allow us sufficient time to prepare

### 3.4. Centrality Indices and Bridge Expected Influence

Figures 5 and 6 display the centrality indices (strength, closeness, and betweenness) and bridge expected influence for the total sample and the three subgroups. Tests indicated that strength served as the dominant centrality metric (correlation coefficients > 0.5). In the overall network, symptom E14 emerged as the most critical node, exhibiting the highest strength (rs = 15.928) and closeness (rc = 0.011), as well as the highest bridge expected influence (rs = 11.282), followed by E13 (rs = 11.199) and E12 (rs = 10.987) in bridge strength. Within the subgroups, the core and bridging symptoms varied substantially. In the C1 network, M11 possessed the highest strength centrality (rs = 7.486), whereas E12 exhibited the highest bridge expected influence (rs = 4.823). In the C2 network, F5 displayed both the highest strength (rs = 3.405) and closeness (rc = 0.003), with E8 identified as the strongest bridge symptom (rs = 2.866). Finally, in the C3 network, M3 possessed the highest strength centrality (rs = 5.654), and F2 emerged as the symptom with the highest bridge expected influence (rs = 4.060).

FIGURE 5Centrality indices of the total sample and three subgroups (A represents the total sample; B/C/D represents the C1/C2/C3 subgroup). (A) Centrality indices of the total sample. (B) Centrality indices of the C1 sample. (C) Centrality indices of the C2 sample. (D) Centrality indices of the C3 sample.

**Figure:**
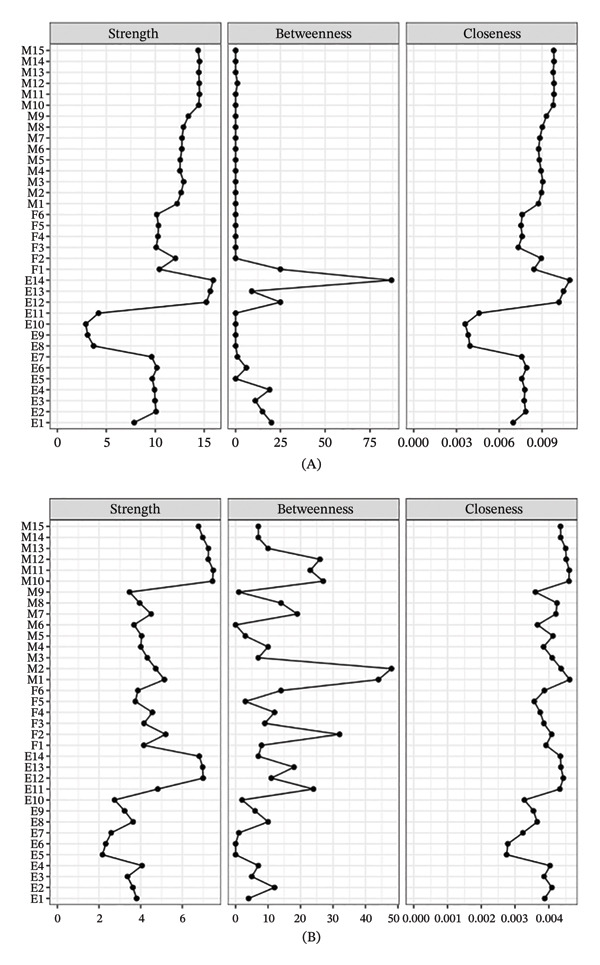

**Figure:**
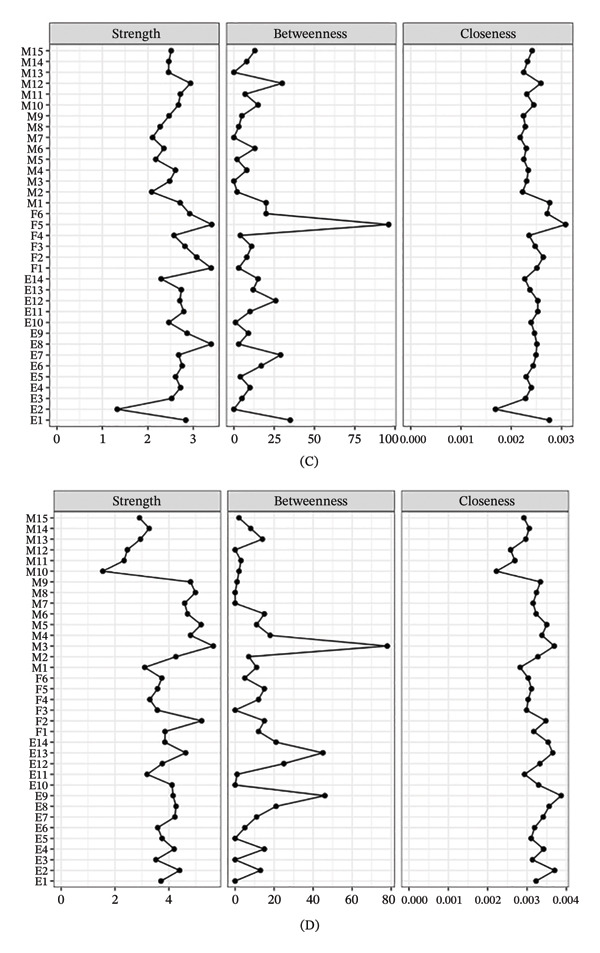

FIGURE 6Bridge strength of the total sample and three subgroups (A represents the total sample; B/C/D represents the C1/C2/C3 subgroup). (A) Bridge strength of the total sample. (B) Bridge strength of the C1 sample. (C) Bridge strength of the C2 sample. (D) Bridge strength of the C3 sample.

**Figure:**
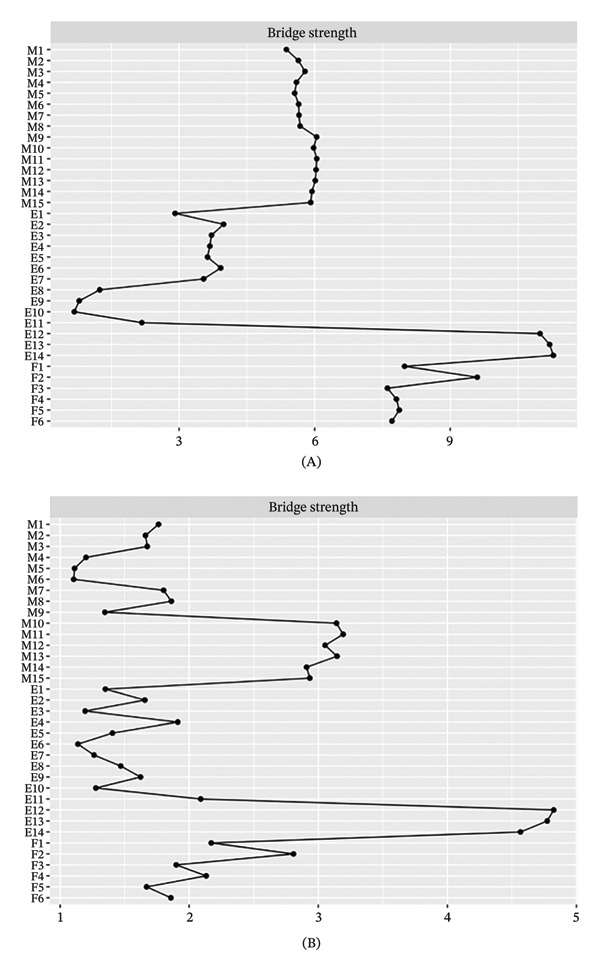

**Figure:**
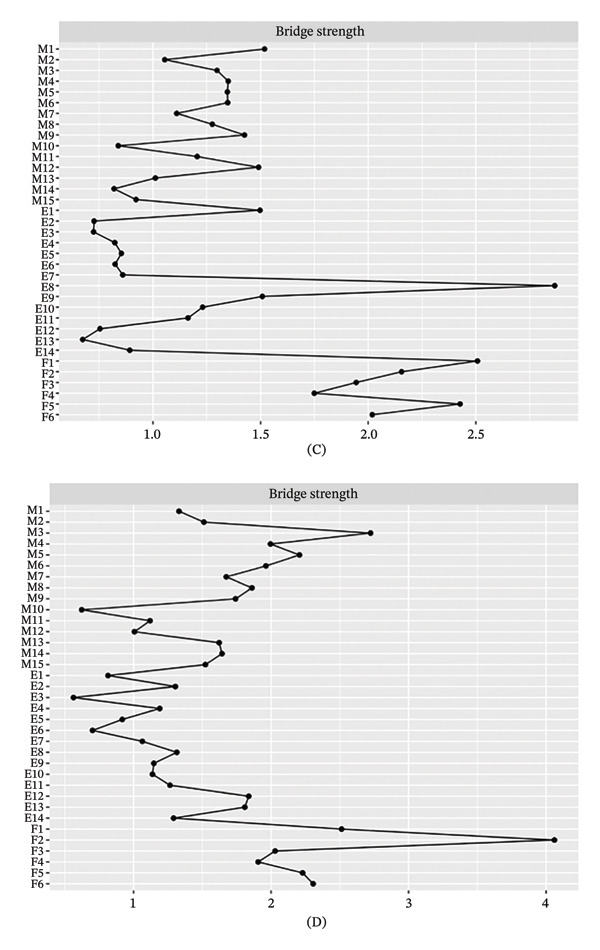

### 3.5. Accuracy and Stability of the Network

**FIGURE 7:**
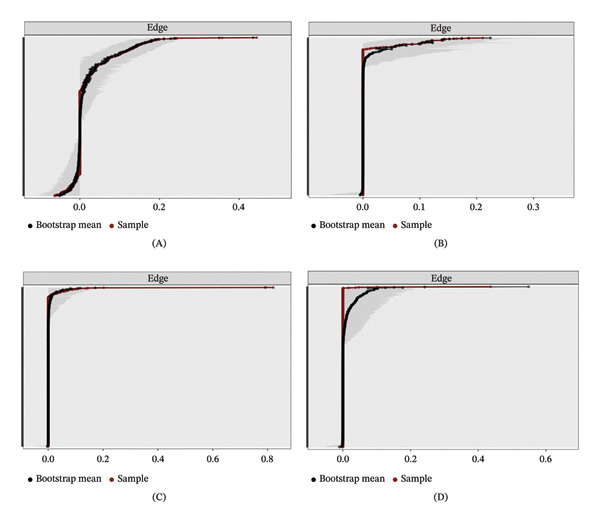
Edge weight accuracy estimation (A represents the total sample; B/C/D represents the C1/C2/C3 subgroup). (A) Accuracy of the total sample. (B) Accuracy of the C1 sample. (C) Accuracy of the C2 sample. (D) Accuracy of the C3 sample.

**FIGURE 8:**
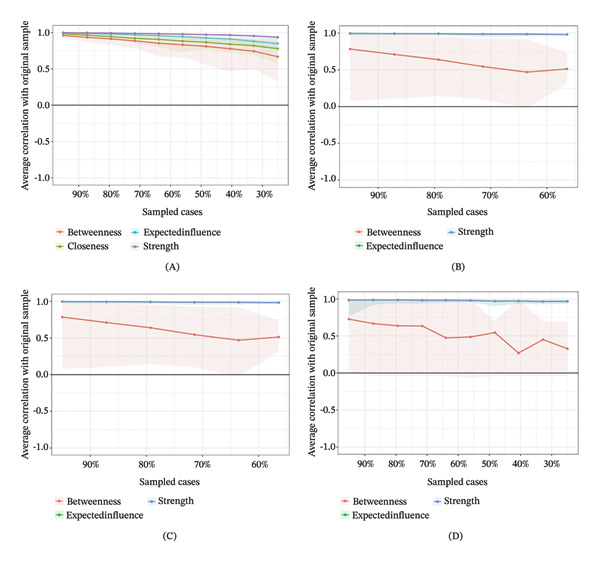
Stability of the total sample and three subgroups (A represents the total sample; B/C/D represents the C1/C2/C3 subgroup). (A) Stability of the total sample. (B) Stability of the C1 sample. (C) Stability of the C2 sample. (D) Stability of the C3 sample.

This study employed nonparametric bootstrap procedures to assess the accuracy and stability of the estimated networks. First, we calculated 95% confidence intervals (CIs) for all edge weights. Figure 7 presents the edge weight results, showing substantial overlap between the bootstrap CIs and the original estimates, indicating good accuracy of the edge weights. Second, we specifically evaluated the edge‐level stability for the C3 subgroup (the smallest profile, *n* = 140) by examining the proportion of bootstrap samples in which each original edge remained nonzero. Remarkably, all 10 core edges in the C3 network were retained in 100% of the 1000 bootstrap resamples, demonstrating excellent edge‐wise reproducibility. Third, the stability of centrality metrics was quantified using the CS coefficient. A higher CS coefficient indicates that node centrality remains similar after removing a larger proportion of the sample, signifying stronger stability. It is generally considered that a CS coefficient greater than 0.25 is acceptable. As shown in Figure 8, the CS coefficient for the overall sample is 0.75, and all CS coefficients for the three subgroups exceed 0.25 (with Subgroup C3 at 0.26). These results confirm that the network structure, including the relatively small C3 subgroup, is sufficiently reliable.

### 3.6. Potential Profile Type‐Network Comparison

**FIGURE 9:**
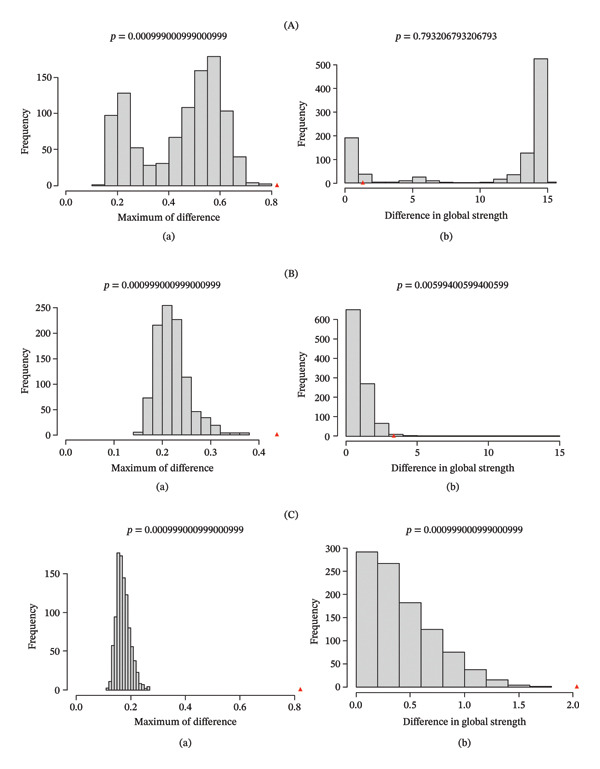
Comparison of network properties among three subgroups (the left‐hand panel shows the results of the network invariance test; the right‐hand panel shows the results of the global invariance test). (A) Comparison of networks properties between C1 and C2. (B) Comparison of networks properties between C1 and C3. (C) Comparison of networks properties between C2 and C3.

NCT results indicate that, based on network invariance tests, statistically significant differences exist between C1 and C3 networks (*M* = 0.437, *p* < 0.001), between C1 and C2 networks (*M* = 0.821, *p* < 0.001), and between C2 and C3 networks (*M* = 0.821, *p* < 0.001). Based on the global strength invariance test, statistically significant differences were found between C1 and C3 networks (*S* = 0.347, *p* = 0.006) and between C2 and C3 networks (*S* = 2.036, *p* < 0.001), but no significant difference was observed between C1 and C2 networks (*S* = 1.312, *p* = 0.793) (Figure 9). Compared to the C2 network, both the C1 and C3 groups exhibited higher network density, with the C3 network showing the strongest edge thickness. Additionally, significant negative correlations were observed between certain edges in the C1 and C2 networks.

### 3.7. Computer Simulation Intervention

To explore potential intervention strategies, we employed a computer simulation, sequentially modeling the effects of aggravating or alleviating each node on the total symptom burden across the total sample and three subgroups, as shown in Figure 10. It is important to note that these results represent model‐based predictions rather than empirical tests. Symptoms within the blue effective zone (corrected *p* < 0.05) were identified as theoretically sensitive targets. In the total sample, simulating the activation of node M14 (“I complete a lot of valuable work”) yielded the strongest predicted deterioration in the total score (Δ = +1.01, +4.20%). Conversely, simulating the inhibition of node E4 (“When facing patients, I project the required professional demeanor without altering my actual inner feelings”) produced the greatest predicted reduction (Δ = −1.74%, −7.24%), suggesting it as a promising candidate for future empirical research. Subgroup simulations revealed that inhibiting symptom E8 (“The hospital evaluates my work attitude as part of performance reviews”) consistently generated significant predicted improvements across all three profiles (total score reductions of 4.00%–7.50%), highlighting its potential as a cross‐contextual focus. Full simulation details are available in Supporting Information 6.

FIGURE 10Optimal intervention targets for the total sample and three subgroups (A represents the total sample; B/C/D represents the C1/C2/C3 subgroup). (A) Optimal intervention targets of the total sample. (B) Optimal intervention targets of the C1 sample. (C) Optimal intervention targets of the C2 sample. (D) Optimal intervention targets of the C3 sample.

**Figure:**
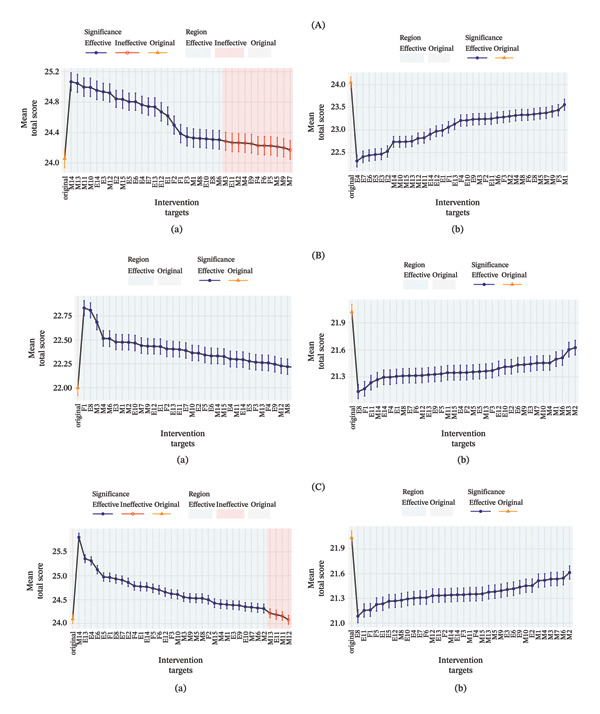

**Figure:**
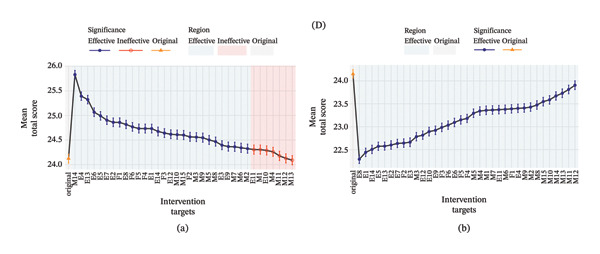

## 4. Discussion

Previous studies have predominantly employed quantitative analytical methods; however, significant interindividual differences exist, often resulting in identical total scale scores but divergent item scoring patterns. Therefore, when analyzing nurses’ emotional labor, it is imperative to fully account for individual heterogeneity. This study, grounded in an individual‐centered perspective, pioneers the application of LPA to identify three distinct emotional labor patterns among Chinese oncology nurses: C1, labeled as the “Efficient Type‐Proactive Regulation Group” (39.9%): high usage of all three strategies (surface acting, deep acting, and emotional expression); C2, termed the “Stressed Type‐Natural Expression Group” (44.9%): moderately high use of surface acting and emotional expression strategies, moderately low use of deep acting; and C3, identified as the “Exhausted Type‐Dysregulated Regulation Group” (15.2%): most preferred use of emotional expression strategies, followed by deep acting strategies, with the lowest use of surface acting strategies.

This study found that nurses’ emotional labor profiles were significantly shaped by demographic and workplace variables. Our finding indicates that nurses with less clinical experience (e.g., younger age, fewer years of service, junior titles), higher work intensity (e.g., frequent night shifts, high‐stress departments like ICU/emergency), and higher neuroticism were more likely to fall into the maladaptive C3 profile. These findings, consistent with prior studies [41, 42], confirm that demographic/workplace factors shape emotional labor patterns. Therefore, when developing emotional labor management strategies, nursing administrators should fully consider nurses’ individual characteristics and work contexts. By rationally allocating workloads, enhancing support systems, and establishing incentive mechanisms, they can alleviate the emotional burden on oncology nurses, thereby promoting their mental well‐being [43].

The correlation analysis, controlling for demographic variables, revealed distinct associations between the three emotional labor strategies and the outcomes. Surface acting was positively correlated with change fatigue but negatively with occupational burnout. Emotional expression showed a positive correlation with change fatigue and a negative correlation with burnout. Deep acting was positively (though nonsignificantly) associated with burnout and negatively with change fatigue, aligning with prior findings [44–46]. Interpreted through COR theory, surface acting requires sustained conscious effort, explaining its link to higher change fatigue. Its negative association with burnout may reflect short‐term suppression of exhaustion, though prolonged use risks resource depletion. Both emotional expression and deep acting also demand volitional effort but can foster self‐authenticity, efficacy, and positive social feedback, offering potential resource compensation. This is reflected in emotional expression’s positive tie to personal accomplishment (a reverse‐coded burnout dimension) and its association with change fatigue. Notably, the positive correlation between deep acting and burnout was not statistically significant. This may be because burnout is multidimensional, encompassing both positive (e.g., personal accomplishment) and negative (e.g., emotional exhaustion) aspects. Future research should therefore analyze burnout subdimensions separately to clarify the specific mechanisms linking emotional labor strategies to change fatigue.

Additionally, nurses experiencing different levels of emotional labor intensity (low—C1 group, moderate—C2 group, and high—C3 group) exhibited significant differences in their burnout and change fatigue scores. Specifically, both burnout and change fatigue scores followed a pattern of C3 > C2 > C1, suggesting that nurses’ levels of burnout and change fatigue may correlate with the intensity of their emotional labor strategies. Notably, the ability to regulate emotional strategies may serve as a key protective factor in mitigating high‐intensity emotional labor and its potential negative consequences, such as burnout and change fatigue. Therefore, providing ongoing education and professional development opportunities is particularly important [47]. Such interventions aim to guide nurses toward more rational and effective use of emotional labor strategies by enhancing their professional knowledge and strengthening their psychological resilience traits. This targeted approach seeks to reduce burnout and change fatigue levels among nurses across different emotional labor intensity groups, thereby achieving precision intervention.

Network analysis identified E14 (“effort to feel and embody required emotions for patients”) as the node with the highest strength centrality in the overall emotional labor–change fatigue–occupational burnout network, acting as a prominent hub with the strongest direct associations to other nodes [48]. Although highly central nodes are theoretically considered potential intervention targets due to their strong connectivity—as supported by prior studies (e.g., targeting grief in lung cancer survivors is associated with lower overall symptom severity; addressing inferiority feelings in HIV patients corresponds to milder multiple symptoms [49, 50]) —these findings should be interpreted with caution, as they are based on cross‐sectional data and do not imply causality. This study suggests a potential dual‐association pattern for E14: a positive correlational pattern (associated with F6 and M13, potentially reflecting network stability) and an exhaustion‐related associative pattern (structurally linked to F2 and M9, consistent with COR theory). Distinct psychological patterns were observed across three profiles: C1—Efficacy Barrier Type, C2—Environmental Penetration Type, and C3—Physiological Exhaustion Type. These observations suggest that intervention strategies might be more effective if tailored to each profile’s specific network topology, e.g., focusing on efficacy for C1 or environmental stressors for C2. Notably, F1 in C2 had the lowest centrality (rs = 3.393), placing it at the periphery of the cross‐sectional network. Theoretically, intervening on these peripherally associated symptoms might be associated with lower connectivity of highly central nodes such as E14 or M3; however, temporal ordering and causal processes cannot be inferred from cross‐sectional data.

Bridge symptoms represent nodes that exhibit strong associations across distinct clusters within a network, potentially reflecting the interconnectedness of multidimensional constructs [51]. In psychological networks, these nodes are often identified using bridge strength, which quantifies the sum of the absolute weights of all edges connecting a node to symptoms in other clusters [36]. Given the ongoing methodological debates regarding the reliability and replicability of centrality indices, interpretations of these metrics must be made with caution. Consistent with our previous justification, we focused on bridge strength as a relatively stable indicator of intercluster connectivity, though it strictly represents statistical associations rather than causal influence. Our results identified E14 (“effort to feel and embody the required emotions for patients”) as the node with the highest bridge strength in the total sample, suggesting it shares the most robust correlations across the emotional labor, change fatigue, and occupational burnout clusters. Although this strong association highlights that deep acting is closely intertwined with mental health indicators, the cross‐sectional nature of our design strictly precludes identifying E14 as a definitive “precursor” or causal driver. These findings reflect simultaneous co‐occurrences rather than longitudinal development. The observed heterogeneity in bridge symptoms across profiles (e.g., E12 in C1, E8 in C2, and F2 in C3) points to diverse associative patterns within the nursing cohort, reflecting different structural organizations of symptoms rather than sequential vulnerabilities. Theoretically, bridge symptoms are regarded as potential focal points for understanding how symptoms in one domain might co‐occur with those in another [52]. In this study, the connectivity of E14 aligns with the framework of COR theory, generating hypotheses for two potential correlational patterns: 1) a potential “resource exhaustion” pattern where E14 is strongly correlated with change fatigue (F2) and emotional exhaustion (M3) and 2) a possible “resilience suppression” pattern where E14 is linked to variables indicating insufficient resource regeneration. However, we emphasize that the current cross‐sectional network structure does not establish temporal or mechanistic direction. Consequently, this theoretical integration should be strictly framed as hypothesis‐generating rather than definitive evidence of underlying mechanisms. We explicitly caution against interpreting these static associations as dynamic “cascades” or “catalytic processes.”

Furthermore, after controlling for all covariates, network invariance tests revealed statistically significant differences in network structure among the C1–C3, C1–C2, and C2–C3 groups (*p* < 0.001). Network density analysis showed that the C1 subgroup had the highest density (0.37), followed by C3 (0.32) and C2 (0.26). Existing research indicates that network density is more sensitive than symptom severity and can serve as an indicator for distinguishing population subtypes and predicting long‐term treatment outcomes [53]. Given the small sample size of the C3 subgroup, we assessed its network stability using bootstrap analysis: The centrality CS coefficient was 0.26 (meeting the acceptable threshold of > 0.25), and all 10 core edges were retained in 100% of the resampling runs, supporting its acceptable robustness. However, due to insufficient statistical power resulting from the limited sample size, the findings regarding the C3 subgroup should best be regarded as exploratory results. Our research team will initiate a prospective cohort study to extend the follow‐up period, track the dynamic evolution of network density in nurses’ emotional labor characteristics, and analyze the dose–response relationship between the network density of the “emotional labor–change fatigue–burnout” network and quality‐of‐life indicators.

Our in silico network simulation revealed that perturbing the superficially positive symptom M14 (“I complete a lot of valuable work”) predicted the strongest increase in overall network activation in the total sample (Δ = +1.01) and the C3 subgroup (Δ = +1.80). This structurally reflects an “overcommitment” pattern—tying self‐worth excessively to work achievements—which is strongly linked to resource depletion, particularly in the psychologically vulnerable C3 subgroup. Meanwhile, F1 (“The organization has introduced too many changes…”) predicted the highest activation in Subgroups C1 and C2, indicating that sustained organizational change remains a key structural risk even for groups with sufficient psychological resources. Regarding simulated alleviating interventions, perturbing E4 (“When facing patients, I project the required professional demeanor without altering my actual inner feelings”) predicted the most substantial reduction in network activation in the total sample. Theoretically, this suggests individual emotion regulation training could decouple emotional demands from internal resource depletion. Concurrently, simulated interventions on E8 (“The hospital evaluates my work attitude…”) consistently predicted reduced activation across all subgroups (C1: Δ = −0.88; C2: Δ = −0.96; C3: Δ = −1.82). This robust cross‐context effect suggests that optimizing organizational performance evaluations could serve as a broad structural prevention strategy. In summary, M14 (endogenous overcommitment) and F1 (exogenous change pressure) act as central associative risk factors, whereas E4 (individual emotion regulation) and E8 (organizational evaluation systems) represent key potential targets for intervention. However, we emphasize that temporal ordering and causal processes cannot be inferred from cross‐sectional data. Theoretically perturbing a node simulates a targeted intervention to alter its baseline susceptibility; thus, these results are generative, model‐derived predictions rather than empirical observations. Although targeting these structural nodes provides a theoretical framework for occupational mental health, their actual clinical efficacy must be rigorously validated through future longitudinal studies and randomized controlled trials.

## 5. Limitations

Although this study presents innovative findings, it has limitations. First, the cross‐sectional design precludes any causal inferences regarding the relationships between emotional labor and burnout; the observed network structures represent statistical associations rather than temporal mechanisms. Second, the data collection relied on online self‐reported measures, which may be subject to recall bias or social desirability bias. Future studies could incorporate objective measures or adopt mixed‐methods approaches. Third, the sample was drawn from a single Chinese cancer hospital, limiting the generalizability of results to nurses in other clinical settings or cultural contexts. Fourth, the simulation‐based intervention analysis relies inherently on mathematical model assumptions rather than empirical validation; future longitudinal studies or randomized controlled trials are required to clinically verify these predicted outcomes. Finally, regarding methodology, although the stability of the C3 subgroup network was acceptable, the small sample size calls for validation in larger prospective cohorts. Moreover, our method treats the membership of LPA‐based feature sets as fixed values, without formally accounting for classification uncertainty. Although high classification quality minimizes this risk, future studies could adopt approaches that account for classification errors and explore neurophysiological indicators (such as salivary cortisol) to deepen the biological understanding of core symptoms.

## 6. Conclusion

In conclusion, this study used person‐centered network analysis combined with computer simulation to systematically examine the complex associative patterns among emotional labor, change fatigue, and occupational burnout in Chinese oncology nurses. Three distinct emotional labor profiles were identified: efficient proactive regulator (C1), stressed natural expressor (C2), and exhausted dysregulated regulator (C3). Network analysis indicated that deep acting (E14) served as a pivotal hub and bridge symptom, which may be linked to theoretical patterns of resource depletion and compensation failure. Computer simulations identified potential core leverage points: M14 as a destabilizing factor (associated with over‐effort cognition), F1 as a risk source for C1/C2 (reflecting external organizational pressures), E4 as a promising target for the total sample (relevant to the resource depletion framework), and E8 as a cross‐profile robust target (associated with external evaluation pressure). Differences across profiles underscore the necessity for personalized interventions. These findings generate testable hypotheses regarding emotional labor mechanisms and provide an evidence base for developing targeted management strategies through individual skill development and organizational system optimization to manage burnout and foster sustainable care environments.

## Author Contributions

Hang Gao and Xiaoli Sun were responsible for conceptualizing the research and drafting the initial manuscript. Danwen Zheng and Hejia Chen collected the records and conducted statistical analysis. Jianwen Hou contributed to both the writing and revision of the article. Xinyan Yu oversaw the overall quality of the research process.

## Funding

This study was supported by a grant from the 2021 Zhejiang Province Medical and Health Science and Technology Program (No. 2021KY591) and the 2023 Zhejiang Province Medical and Health Science and Technology Program (No. 2023KY568).

## Disclosure

All the authors read and approved the final version of the manuscript.

## Conflicts of Interest

The authors declare no conflicts of interest.

## Supporting Information

Additional supporting information can be found online in the Supporting Information section.

## Supporting information


**Supporting Information** Supporting Table S1: This table contains two sections: Table 1‐A presents a comparison of BIC values for four common covariance structure models (VVV, VVI, VEI, and EII) and Table 1‐B presents the posterior probability matrix reflecting the results of the three‐class classification; Supporting Table S2: This table contains the detailed edge weights and centrality measures for the symptom network of the total sample; Supporting Table S3: This table provides the detailed edge weights and centrality measures for the symptom network of the C1; Supporting Table S4: This table provides the detailed edge weights and centrality measures for the symptom network of the C2; Supporting Table S5: This table provides the detailed edge weights and centrality measures for the symptom network of the C3; and Supporting Information 6 provides full simulation details.

## Data Availability

The original dataset collected and analyzed in this study may be obtained from the corresponding author upon reasonable request.
