# Supplementary material for: Profiles and Networks: A Person‐Centered Analysis of Emotional Labor, Burnout, and Change Fatigue Among Chinese Oncology Nurses
Source: J Nurs Manag. 2026 Jul 12;2026:4262055. doi: 10.1155/jonm/4262055 (PMC13358210; doi:10.1155/jonm/4262055)
Supplement: Supplementary file 1 — Supporting Information Supporting Table S1: This table contains two sections: Table 1‐A presents a comparison of BIC values for four common covariance structure models (VVV, VVI, VEI, and EII) and Table 1‐B presents the posterior probability matrix reflecting the results of the three‐class classification; Supporting Table S2: This table contains the detailed edge weights and centrality measures for the symptom network of the total sample; Supporting Table S3: This table provides the detailed edge weights and centrality measures for the symptom network of the C1; Supporting Table S4: This table provides the detailed edge weights and centrality measures for the symptom network of the C2; Supporting Table S5: This table provides the detailed edge weights and centrality measures for the symptom network of the C3; and Supporting Information 6 provides full simulation details. [file JONM-2026-4262055-s001.zip › Supplementary Table 1(Supplementary Word 1. Supplementary Data for Potential Profile Models).doc]

Supplementary Table 1-A Model Comparison Results

| Model | G=1 | G=2 | G=3 |
| --- | --- | --- | --- |
| VVV | 36820.45 | 34210.88 | 33285.40 |
| **VVI** | **38884.77** | **33819.77** | **33040.62** |
| VEI | 39120.33 | 34325.64 | 33198.75 |
| EII | 40558.62 | 35442.19 | 33876.43 |

EII=equal variances, covariances fixed to 0 (spherical); VEI=varying variances, covariances fixed to 0 (diagonal, equal shape); VVI=varying variances, covariances fixed to 0 (diagonal, varying shape); VVV=varying variances and unrestricted covariances (ellipsoidal). Lower BIC indicates better model fit.

Supplementary Table 1-B. Posterior Probabilities

| **Assigned Profile** | **Predicted Profile 1** | **Predicted Profile 2** | **Predicted Profile 3** |
| --- | --- | --- | --- |
| Profile 1 | **0.935** | 0.065 | <0.001 |
| Profile 2 | 0.055 | **0.944** | 0.001 |
| Profile 3 | <0.001 | 0.011 | **0.999** |
